# Medical Society of Paris—Transactions

**Published:** 1830-10-01

**Authors:** 


### MEDICAL SOCIETY OF PARIS.

A NEW Journal has started in Paris, entitled "MEDICAL TRANSACTIONS," in which are published the transactions of the SOCIÉTÉ DE MÉDÉCINE of that metropolis, and which promises to be more than usually interesting, by giving a fair and ungarbled account of the *viva voce* discussions in that assembly. The reports, in the present case, are drawn up officially by the secretary of the society—M. Gendrin, and, therefore, the fidelity of them is guaranteed in the most satisfactory manner. They will furnish us with an article from time to time, which we hope will not be found void either of interest or instruction.

#### I. TREATMENT OF PERIPNEUMONY BY LARGE DOSES OF EMETIC TARTAR.

M. Thealier made a verbal report of the medical constitution of the last quarter of 1829, as observed in the department of the INDRE ET LOIRE, and read before the Medical Society of Tours. The subject of antimony in pulmonic inflammation was broached, and M. Thealier asserted that he had multiplied his observations to a great extent, and could positively affirm that antimony, in large doses, constituted a most important and efficacious remedy for the phlogosis in question. He avers, indeed, that in many cases, where all other means had failed in arresting the progress of the inflammation, and where the lungs or pleura were threatened with fatal disorganization, the tartrite, in large doses, put a stop to the ravages of the disease. There can be no doubt that antimony is a powerful auxiliary to the lancet in pulmonary, as well as in many other inflammations.

#### II. MYSTERIOUS EXPECTORATION OF PUS—HEPATIC ABSCESS, &c.

A case related to the society by M. Merat occasioned a very warm discussion among the members, and, as usual, drew forth many curious facts and observations. The original case was as follows:—A young lady expectorated twice a day, for a long time, a large quantity of purulent matter, of a very fetid odour. The expectoration occurred early in the morning, and again at two o'clock, p.m. The paroxysm, which generally lasted only a few minutes, was preceded by a sense of suffocation—then came on a slight cough, and a discharge of a tumblerful of pus in a few minutes. The chest was examined with the stethoscope, and it was thought that an excavation existed in the posterior and lower portion of the left lung, but they do not appear to have been very positive as to that point. This young lady had been subject to attacks of this kind for some years, and married, contrary to the advice of M. Merat, at an early age, and became pregnant. She got to the eighth month of utero-gestation, having frequently required venesection. The expectoration of purulent matter ceased, and this lady died in two or three days' illness. On examination, (which was unavoidably confined to the thorax) no trace of disease could be found in either the lungs or their coverings. There were no adhesions—no trace, in short, of any malady whatever.

M. Gendrin remarked that, as hepatic abscesses sometimes make their way through the diaphragm, and the matter is evacuated by the trachea, this might be a case of the kind. It is astonishing that, in such a society, no one observed that such a thing was impossible in the present case, where there were no adhesions, nor any breach of structure in the diaphragm, through which the hepatic pus might pass. M. G. related the case of a lady who had suffered for several years from attacks of purulent expectoration, preceded always by engorgement and tumefaction of the right hypochondrium. The matter was ejected by a combination of vomiting and coughing, after which the region of the liver diminished for two or three weeks, when the symptoms were renewed. This female is still living, and has been seen by Recamier, Dubois, and many other eminent physicians of Paris.

M. Sandras conceived it probable that, in M. Merat's case, the puriform matter came from the stomach—the cough and sense of oppression preceding and accompanying the discharge being no positive proof that the matter came from the lungs. M. Merat himself acknowledged that he now came to a similar conclusion, in which, indeed, we entirely agree. M. Merat declared, however, that the pus, on repeated examinations, was pure, and without the least admixture, apparently, of mucus. Various opinions, supported by cases, were brought forward respecting hepatic abscesses, but these we need not detail.

### III. Operations for internal Strangulations of the Intestines.

M. Sanson informed the Society that he had met with five cases of strangulated hernia, presenting circumstances that were worthy of notice. In the first two cases, the patients had reduced their herniæ before they applied to him, but the symptoms of strangulation continued. There was perceptible to the touch a tumour, immediately above the abdominal ring. An operation was performed. It consisted in laying the ring bare, seizing the tumour in the abdomen, and drawing it into view, when the disentanglement (debridement) of the intestine was readily effected. The patient recovered. The presence, then, of a tumour in the abdomen, after the reduction of a hernia, and the continuance of the symptoms of strangulation, are the signs which should guide the surgeon in the adoption of an operation.

A man was brought to the Hôtel Dieu apparently dying. He had himself reduced a hernia; but all the symptoms continued. Nevertheless, no tumour could be felt in any part of the abdomen. As he was in articulo mortis, it was not judged proper to perform any operation. On examining the exterior of the body after death, M. Sanson and others could not discover any trace of strangulated hernia. Dissection, however, shewed that the tumour constituted by the strangled intestine had receded behind the pubis, instead of remaining above or in the immediate neighbourhood of the ring. A portion of the gut, and also of the epiploon, were gangrened. The fourth case shewed (said M. Sanson) with what caution we should proceed to operate, when the signs and site of strangulation are defective or absent. A man, after a journey, was seized with the symptoms of an internal strangulation. He had constant vomiting, obstinate constipation, inflation, tension, and pain of the abdomen, &c. Stercoraceous matters were at length ejected. This man had had a reducible hernia of long standing, which he had recently put up, and he affirmed that there was nothing unusual in the appearance of the hernia at the time, the truss being applied by him in the usual manner. Warm baths, venesection, and the usual means, produced no relief. A consultation was ordered, and the question of an operation was agitated. The majority decided against the knife. The patient, mean time, continued in the same state till the 15th day from the commencement of the obstruction. A celebrated surgeon was now called in, and, after mature consideration, proposed to operate, although no tumour, or other circumstance, indicated the site of the strangulated gut. The operation was agreed to, and M. Sanson went home to bring the necessary instruments and dressings. When he returned he again carefully examined the abdomen, and found that there was actually less tension and tenderness in the neighbourhood of the ruptured lung than in any other part of the belly. Continuing still the examination, he at length discovered an oblong tumour, deeply seated in the left side of the abdomen, (the hernia was on the right) which appeared to him to be a collection of hardened fæces in the colon. A bougie was introduced into the rectum, but could not be pushed up to any extent, on account of the contracted state of the gut. Oily lavements were then employed, at first without effect, but, by perseverance they brought away some soft fæces. A purgative could not be administered by the mouth, on account of the constant vomiting. The surface of a portion of the thigh was denuded by a blister, and thereon was applied some croton oil. The consequence was an abundant evacuation from the bowels, and instant relief of all the symptoms. The operator would have looked rather foolish had he employed the knife in this case, and been permitted to examine the body after death!

The fifth and last case was also that of a man who evinced all the usual signs of strangulation. The hernia was external, and the operation was performed. When the ring and the sac were divided, the enclosed gut presented nothing particular, and M. Sanson returned the bowel, but found great difficulty in this part of the operation. He found the intestine accumulate above the ring, and therefore introduced his finger into the canal, but could feel no obstruction. He perceived, however, that the finger did not penetrate into the cavity of the abdomen. He incised the ring still farther, and then discovered the cause of the accumulation of intestine. Above the ring he found the hernial sac was dilated into a pouch, which communicated with the general cavity of the abdomen by means of a narrow aperture. This aperture was obliged to be enlarged before the complete reduction of the intestine could be effected. M. Gerdy related to the society a case almost exactly similar, which had occurred in his own practice, and required a similar operation.

IV. Case of an apparent Aneurismal Tumour—Hepatitis—Insidious Hydrothorax.

M. Merat related the case of a man who was suddenly affected, after a straining exertion in moving some furniture, with a pulsating tumour in the neck, over the course of the carotid artery. Little attention was paid to this swelling till it became painful, when he applied to M. Merat for assistance. The pulsation and the site of the tumour induced M. Merat to conclude that it was an aneurism of the carotid. He advised the application of ice, and under this remedy the tumour diminished in size, and ceased to pulsate; but the patient could not bear the ice, and it was discontinued. The tumour remained stationary for three weeks. M. Dubois examined it, and considered it as aneurismal, but arrested in its progress by the application of the ice. Nevertheless the tumour exhibited a doughy feel, and an irregular circumscription, which induced doubts in the minds of the members of the society, when the patient was presented at a former meeting. Shortly after this the patient evinced signs of acute hepatitis, with an enlargement of the liver below the false ribs. This was attributed to his having taken cold while applying the ice to his neck. The symptoms became more intense—the right hypochondrium swelled, as did that side of the thorax—the respiration became dull in the right lung—the liver was found to descend far below the ribs, and there was excruciating pain extending to the loins. The most active antiphlogistic treatment was employed; but without success. The sense of suffocation increased, and the patient succumbed.

On dissection, by M. Merat and M. Deville, the right side of the chest was found to contain more than eight pints of yellow serum, which had pressed back the lung, and by depressing the diaphragm made the liver to descend below the ribs, and appear enlarged. The pleura shewed no signs of inflammation, and all the thoracic viscera were sound. The cervical tumour was then examined, and found to consist of a mass of glands, the size of an egg, seated over the arteria innominata, at the origin of the right carotid. The coverings of this tumour were highly injected, and discharged much blood when cut into. It is remarkable that till within eight days of his death this man had been able to pursue his usual avocations out of doors. The cause of the hydrothorax is very mysterious, there being no inflammation of the pleura. The case shews how men may be deceived by the appearance of pulsating tumours in the neck. We can have no doubt that the carotid artery has been tied more than once in this country, without any necessity for such an operation!

